# Light‐dependent niche differentiation in two mixotrophic bacterivores

**DOI:** 10.1111/1758-2229.13071

**Published:** 2022-05-04

**Authors:** Robert Fischer, Julia Kitzwögerer, Robert Ptacnik

**Affiliations:** ^1^ AquaScale, WasserCluster Lunz Biologische Station GmbH Lunz am See Austria; ^2^ Austrian Biotech University of Applied Sciences, Biotech Campus Tulln Tulln an der Donau Austria

## Abstract

Mixotrophy usually is considered with respect to the advantages gained and the associated trade‐offs of this form of nutrition, compared to specialized competitors, strict photoautotrophs and heterotrophs. However, we currently have an incomplete understanding of the functional diversity of mixotrophs and the factors controlling niche differentiation in different mixotrophic species. Here we experimentally studied the light‐dependent niche differentiation in two chrysophyte species. We show that the newly isolated *Ochromonas* sp. is an obligate phototroph and possibly an obligate mixotroph. In contrast, *Poterioochromonas malhamensis* is a facultative mixotroph; photosynthesis and heterotrophy in this species represent substitutable routes of resource acquisition. We further hypothesize that the variable plasticity in the considered traits of the here tested species may result in different niche differentiation with regard to a vertical light gradient. *Ochromonas* sp. should perform better in stable stratified surface water layers, where light is available, but prey abundances might be low. However, *P*. *malhamensis* should be able to also successfully grow in deeper water layers, benefiting from higher bacterial production. This study represents a first step towards understanding competition between mixotrophs engaging in different physiological strategies, and consequently their potential co‐occurrence due to niche differentiation.

## Introduction

Mixotrophy in protists, the combination of photoautotrophy and heterotrophy, can be categorized in different ways. Mitra *et al*. (2016) recently defined protists that have an innate capability for C‐fixation and phagocytosis as ‘Constitutive Mixotrophs’, which is the group we here will consider. Specifically, we focus on those mixotrophs that ingest bacteria, mixotrophic bacterivores.

Mixotrophy occurs in almost all protist ‘phytoplankton’ groups, with diatoms being the only true exception (Flynn *et al*., 2012), though only for less than 150 species clear evidence for mixotrophy has been obtained (Leles *et al*., 2018). This low number is caused by the difficulties to detect mixotrophy in individual species in phytoplankton communities in the field and because it is challenging to establish monocultures in the lab, due to our incomplete understanding of the specific requirements of individual species. Nonetheless, in the past decades experimental and conceptual studies on mixotrophy revealed a number of costs and benefits, i.e. trade‐offs, associated with this form of nutrition. The ability to engage in photosynthesis and (phago‐) heterotrophy requires investments in both cellular machineries (Raven, 1997). These energetic costs may lower the resource use efficiency of mixotrophic organisms and hence may lower the overall performance, i.e. a lower maximum growth rate compared to specialized photoautotrophs or heterotrophs (Raven, 1997; Litchman *et al*., 2007; Flynn and Mitra, 2009; Raven *et al*., 2009; Ward *et al*., 2011). Therefore, mixotrophs might be inferior if they compete with strict photoauto‐ or heterotrophs under conditions favourable for either of the specialist strategies. In oligotrophic surface waters, where nutrients are limiting photosynthetic growth but light is available, mixotrophs are able to compete successfully with both specialist groups at the same time, here they have an advantage over photoautotrophs due to the acquisition of nutrients through phagotrophy and might outcompete heterotrophs for prey, since they are able to use light energy (Tittel *et al*., 2003; Fischer *et al*., 2017a). However, mixotrophy has also been described as a strategy to survive (and grow) under light‐limited conditions (Berninger *et al*., 1992; Caron *et al*., 1993), for otherwise primarily photoautotrophic species. This form of mixotrophy is less well supported by empirical studies and may be limited to larger protists in eutrophic systems (Burkholder *et al*., 2008) and to species of the widespread genus *Cryptomonas*, which seem to use a mixotrophic strategy to survive prolonged light limitation (Roberts and Laybourn‐Parry, 1999), but apparently have very low ingestion rates (Tranvik *et al*., 1989).

Mixotrophs in the nano‐plankton size range have been hypothesized and shown to be important in oligotrophic systems (Bird and Kalff, 1986; Mitra *et al*., 2014; Fischer *et al*., 2017a). They often account for the bulk of bacterivory in near‐surface waters, e.g. more than 50% in freshwater (Saad *et al*., 2016) and up to 95% in marine systems (Zubkov and Tarran, 2008), and are dominant members of planktonic communities (Leles *et al*., 2019). Acknowledging mixotrophic nanoflagellates as major bacterivores has far‐reaching implications for our understanding of microbial food webs. In contrast to heterotrophic bacterivores, light mediates the impact of mixotrophic bacterivores (Tittel *et al*., 2003; Ptacnik *et al*., 2016). They may utilize nutrients from bacterivory directly for photosynthesis instead of shunting nutrients back into the dissolved phase (Rothhaupt, 1997), thereby representing a shortcut in the microbial loop (Ptacnik *et al*., 2016). Moreover, the more balanced nutrition of mixotrophs results in a lower and more stable cellular C:N:P stoichiometry across environmental gradients, compared to their specialized heterotrophic and photoautotrophic competitors (Katechakis *et al*., 2005; Moorthi *et al*., 2017; Fischer *et al*., 2017b). This indicates that mixotrophy may buffer stoichiometric constraints for herbivores and enable a more stable secondary production, and hence enhances the transfer of biomass to higher trophic level (Ward and Follows, 2016).

Despite our conceptual understanding on how mixotrophic nanoflagellates are able to successfully compete with their specialized competitors, at present we are largely unaware regarding the phenotypic plasticity of relevant traits within this important functional group (but see Wilken *et al*., 2019). This adds up to the uncertainties of recent modelling approaches involving mixotrophy (Flynn and Mitra, 2009; Berge *et al*., 2017; Leles *et al*., 2018), which struggle with the insufficient empirical basis for model assumptions and parameterizations (Glibert and Mitra, 2022). In order to understand how mixotrophs compete with each other and which factors drive niche differentiation of different mixotrophs in the vertical water column, i.e. in the light gradient, we need to study mixotrophs in a variable environmental context, considering relevant traits and trait‐combinations.

Here we experimentally study two chrysophyte species, *Poterioochromonas malhamensis* and *Ochromonas* sp. The latter is an isolate from the oligotrophic Lake Lunz (Austria) and its mixotrophic abilities are studied for the first time. *Poterioochromonas malhamensis* in contrast is a rather well‐studied species (e.g. Caron *et al*., 1990; Sanders *et al*., 1990; Holen, 2001). Using a continuous culture method (see Supporting Information) to follow growth trajectories, we apply a light gradient (three light levels: ‘low light’, ‘intermediate light’ and ‘high light’; see Supporting Information) in combination with or without addition of dissolved organic carbon (DOC) in order to enhance bacterial (prey) production (see Supporting Information). We explore four hypotheses. First, *Ochromonas* sp., the presumably more phototrophic mixotroph, requires more light, but a lower minimal prey concentration, compared to the more heterotrophic mixotroph *P*. *malhamensis*. Second, *Ochromonas* sp. remineralizes few nutrients, due to efficiently using nutrients from ingested bacteria for photosynthetic growth. The more heterotrophic metabolism of *P*. *malhamensis* implies higher nutrient remineralization. Third, since *Ochromonas* sp. should be more dependent on light we expect overall higher cellular chlorophyll concentrations, compared to *P*. *malhamensis*. Moreover, we expect an inverse relationship between light and cellular chlorophyll concentrations in *Ochromonas* sp., due to photoacclimation. In contrast, we expect chlorophyll concentrations to be independent of light in *P*. *malhamensis*, due to its ability to supplement energetic requirements by bacterivory under low light conditions. Fourth, mixotrophs overall are assumed to have a more stable cellular stoichiometry and lower carbon to nutrient ratios, compared to their strictly phototrophic competitors, due to their more balanced nutrition (Moorthi *et al*., 2017). Within these margins we expect higher C:P ratios under photosynthetic growth (i.e. without glucose addition), compared to mixotrophic growth (i.e. with glucose addition).

## Results and discussion

**Fig. 1:**
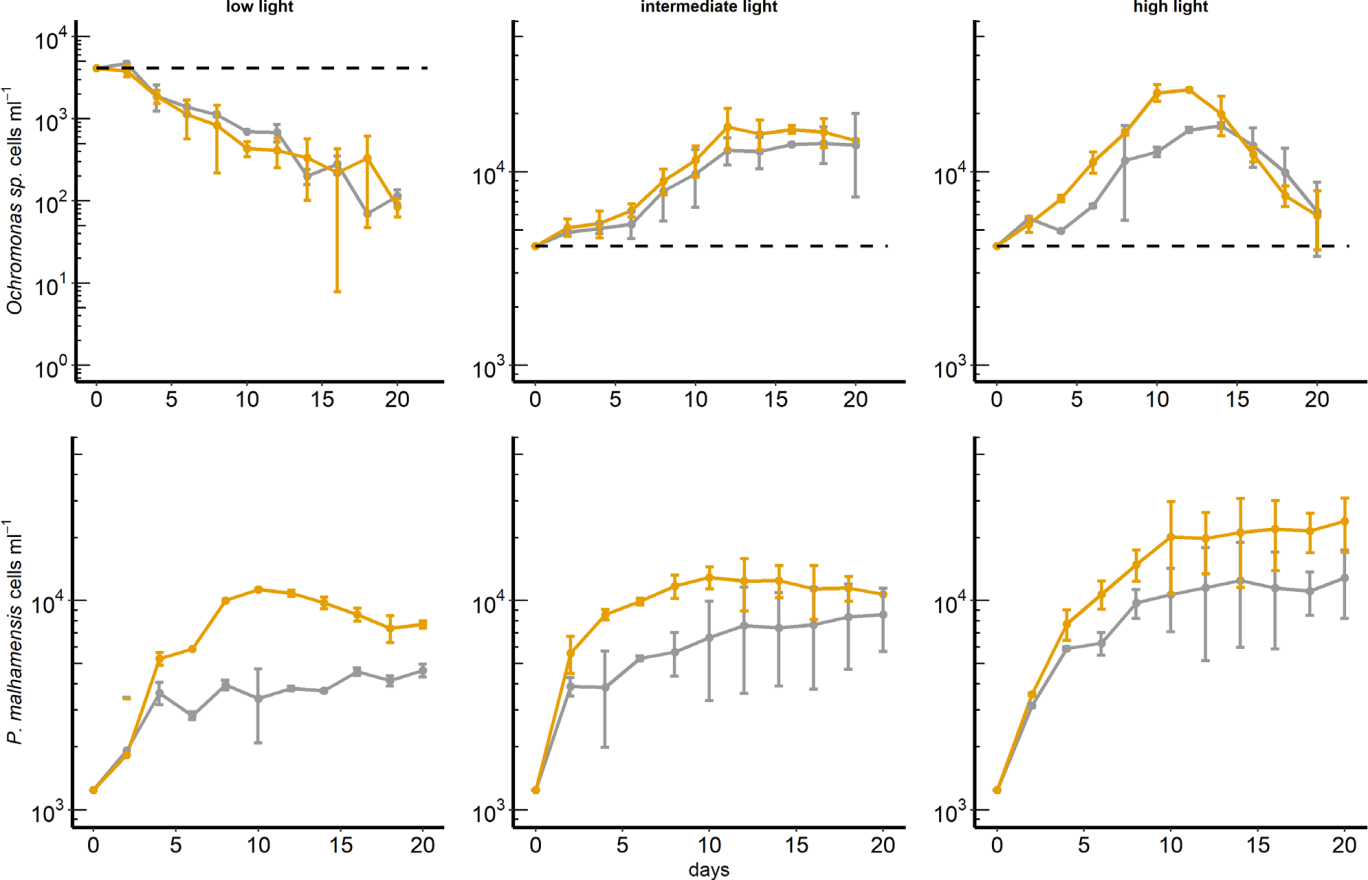
Abundances (cells ml^−1^; calculated average of the two replicates per treatment) of *Ochromonas* sp. (upper panel) and *P*. *malhamensis* (lower panel) over time for three light levels (low light: 10 μmol photons m^2^ s^−1^; intermediate light: 60 μmol photons m^2^ s^−1^; high light: 120 μmol photons m^2^ s^−1^; see Supporting Information) and with glucose addition (yellow), or without glucose addition (grey) respectively. The horizontal dashed line in the upper panel represents the initial abundance of *Ochromonas* sp.

The two mixotrophic chrysophyte species studied here showed different responses to the applied environmental conditions (Fig. 1). We report that the newly isolated *Ochromonas* sp. is an obligate phototroph. This species possibly is an obligate mixotroph, in which photosynthesis and phagotrophy constitute complimentary routes of resource acquisition; however, its obligate requirement for prey has to be experimentally validated, i.e. in axenic cultures. *Poterioochromonas malhamensis* on the other hand appears to be a facultative mixotroph; photosynthesis and heterotrophy in this species represent substitutable routes of resource acquisition. Our experiments did not allow testing the species under strict phototrophic growth conditions, since stock cultures were not axenic, containing unidentified heterotrophic bacteria. Assuming that our categorization of the two mixotrophic species is correct, only the facultative mixotroph *P*. *malhamensis* should be able to sustain growth even under very low natural bacteria abundances, i.e. below threshold concentrations required by other phagotrophic species (Wilken *et al*., 2019). In this situation, *P*. *malhamensis* would rely on photosynthetic C‐fixation and uptake of dissolved nutrients, possibly supplemented by low rates of bacterivory. This has been validated in previous studies on this species (Caron *et al*., 1990; Sanders *et al*., 1990; Holen, 2001).

### Growth trajectories in response to light and DOC addition

In agreement with our first hypotheses *P*. *malhamensis* was able to achieve positive net growth at the lowest irradiance. Abundances of *P*. *malhamensis* increased over time and equilibrated in all treatments and overall showed a strong positive relationship with light (*F*_1,7_ = 33.845, *p* < 0.005), irrespective of the addition of glucose. However, glucose addition resulted in higher, though non‐significantly, abundances at all light levels (*F*_1,7_ = 4.213, *p* = 0.0792).

*Ochromonas* sp. exhibited negative growth rates at the lowest light level (Fig. 1), irrespective of glucose addition, hence *Ochromonas* sp. could not compensate lack of light by bacterivory. In the intermediate light treatments abundances of *Ochromonas* sp. increased (until ca. day 12; Fig. 1) then equilibrated towards the end of the experiment, with higher abundances in +DOC‐treatments. In high light treatments abundances of *Ochromonas* sp. increased towards the mid of the experiment, but then unexpectedly decreased towards the end of the experiment to approximately initial abundances. Based on the determined parameter in the experiment we are not able to provide a reliable explanation for this pattern (but see section, ‘Dissolved inorganic phosphorus’). The initial increase in abundances in the high light treatment, however, was faster in the +DOC‐treatments (peaking at day 10) and lead to higher peak abundances, i.e. the highest abundance reached over the course of the experiment, irrespective of a subsequent decrease. Enhanced bacterial growth led to higher peak cell densities, as long as light was sufficient (intermediate and high light treatments). The fact that *Ochromonas* sp. was unable to grow in the low light treatments, irrespective of glucose addition, suggests that this species is not able to use prey as a source for direct carbon acquisition, or efficiently use DOC for growth respectively. In turn, this suggests that this species preys on bacteria to acquire nutrients or other growth factors respectively. Although it appears very likely, further experiments have to clarify whether these acquired substances are directly used to fuel photosynthetic growth.

In general, glucose addition, and hence increased bacterial production, had a positive effect on the growth of both species. We assume that bacteria were the better competitors for the DOC (glucose), added in low concentrations (see Supporting Information), and hence exclude the possibility that DOC was efficiently used by the protists for growth. Indeed the osmotrophic uptake of dissolved organic substrates in phytoplankton is common; however, bacteria should generally have higher affinities for such substrates. Moreover, the underlying trade‐offs of the mixotrophic nutrition suggest that mixotrophs only show low level of uptake of dissolved organic and inorganic substrates (Flynn and Mitra, 2009). If *Ochromonas* sp. was able to efficiently use the added DOC for growth it also should have been able to grow in the low light treatments. *Poterioochromonas malhamensis* appears to be able to use dissolved organics, however, even high amounts do not increase growth rates, and growth rates on organics in the absence of bacteria prey are very low (Sanders *et al*., 1990). This indicates that this species, albeit fundamentally able to use dissolved organics, is not very efficient in using them for growth, much in contrast to heterotrophic bacteria.

### Dissolved inorganic phosphorus

**Fig. 2:**
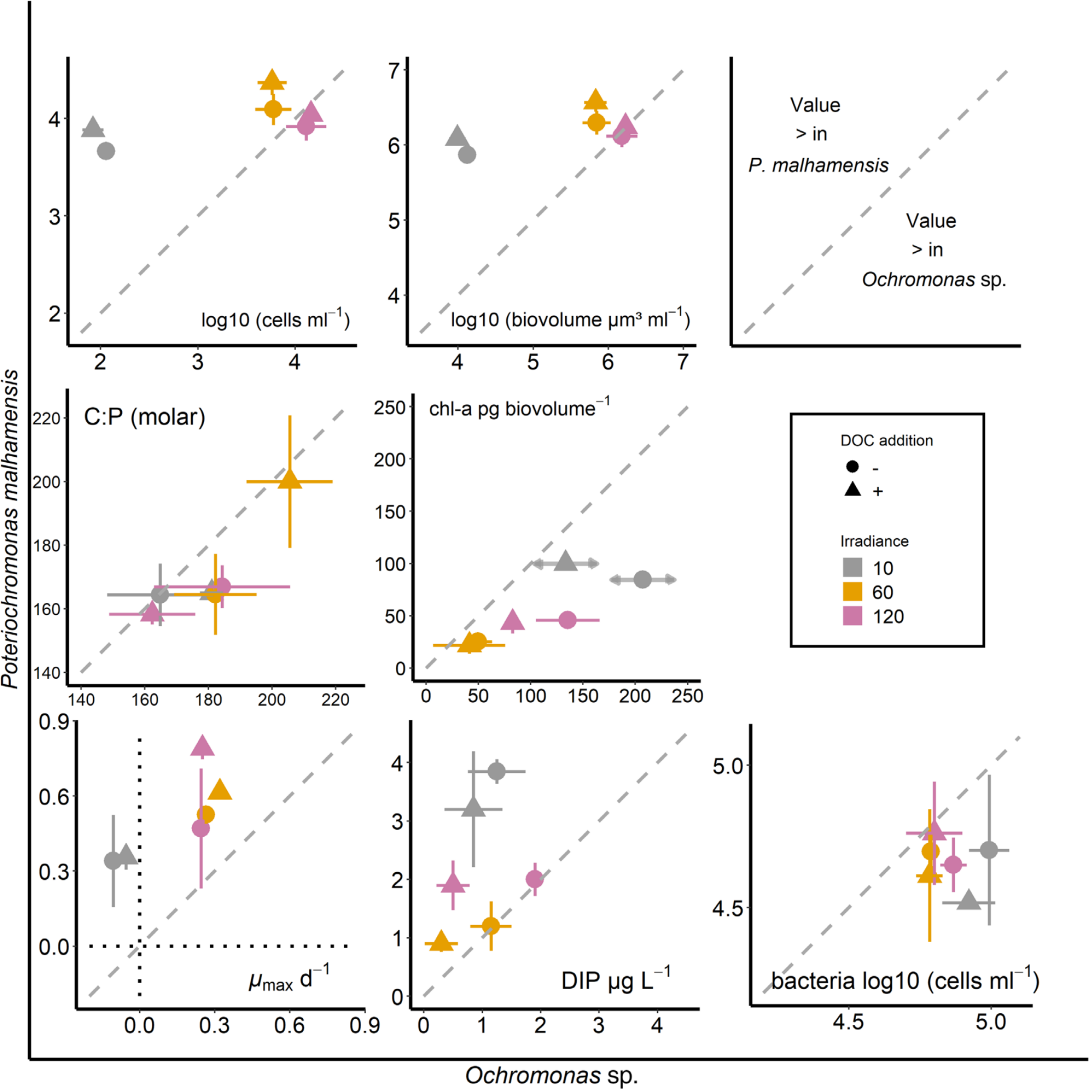
Each plot shows one analysed parameter at the end of the experiment, for both species, with treatments (light level and ±glucose addition) plotted as *xy*‐pairs. The diagonal dashed lines indicate identical values. Values above this 1:1 line indicate that the respective values are higher in *Poterioochromonas malhamensis*, and values below this line indicate that the respective values are higher for *Ochromonas* sp. (as shown in the top right panel). Analysed parameters: protist abundance (cells ml^−1^), protist biovolume (μm^3^ ml^−1^), particulate C:P (molar), chlorophyll‐a (corrected for biovolume; pg biovolume^−1^), DIP (μg L^−1^) and bacteria abundance (cells ml^−1^). Maximum specific growth rates (*μ*_max_ d^−1^) in the lower left panel represent the maximum growth rate during rapid growth in the early stage of the experiment. The dotted lines in this panel indicate zero growth for either species. Symbols indicate the averages of duplicates, crosshairs standard deviations. The grey horizontal arrows in the chlorophyll plot indicate uncertainties regarding the measurements of cellular chlorophyll content of *Ochromonas* sp. in the low light treatments, hence, the shown averages of chlorophyll content at low light represent extrapolated values from linear regression of data for the other two irradiances.

In the *P*. *malhamensis* treatments dissolved inorganic phosphorus (DIP) concentrations (Fig. 2) were inversely related with light (*F*_1,8_ = 45.236, *p* = 0.0001), but independent of glucose addition (*F*_1,8_ = 1.383, *p* = 0.2733). In terms of carbon acquisition and respiration the heterotrophic proportion in the nutrition of the facultative mixotroph *P*. *malhamensis* should be more similar to a strict heterotroph. Consequently, it remineralizes more nutrients, due to respirational losses. Thus, the inverse relationship between DIP and light may indicate for increasing rates of bacterivory with decreasing light, to compensate for decreased rates of carbon fixation.

In agreement with our second hypothesis, DIP concentrations (Fig. 2) across treatments were significantly lower (*F*_1,18_ = 20.276, *p* = 0.0003) in the *Ochromonas* sp. treatments, compared to the *P*. *malhamensis* treatments. This points at higher resource use efficiency in this species. *Ochromonas* sp. efficiently uses nutrients acquired from bacteria prey (and possibly from the dissolved pool) for photosynthetic growth, and hence remineralizes few nutrients, reducing overall concentrations availably in the dissolved pool. In the +DOC treatments bacteria, which generally have higher affinities for dissolved nutrients due to their higher surface to volume ratio, reduced DIP concentrations and were then grazed by *Ochromonas* sp. The combined effect of enhanced bacteria growth and low remineralization of nutrients of *Ochromonas* sp., hence, resulted in significantly lower DIP concentrations in the +DOC treatments (*F*_1,7_ = 10.195, *p* = 0.0152).

Though it remains speculative, low level of nutrient remineralization in *Ochromonas* sp. might also explain the decrease in abundance in the high light treatments. This is a somewhat paradoxical situation, in which the activities of *Ochromonas* sp. produced conditions unfavourable for its own growth (Thingstad *et al*., 1996), low concentrations of dissolved nutrients (P) combined with low bacterial production (due to P, and likely, C‐limitation of the bacteria). In this situation *Ochromonas* sp. has positive net growth until bacteria abundances are reduced below a certain threshold, below which the prey concentrations do not support positive net growth anymore. Beyond this point bacteria are released from grazing; however, their growth is now nutrient limited (and likely energy/C‐limited as well) and they are not able for positive net growth. This could either result in a total washout of the mixotroph or an oscillation with very low frequency respectively. However, we cannot exclude the possibility that the decrease of abundances could have been caused by some other, not determined parameter.

### Chlorophyll‐a content

For comparisons between species, chlorophyll concentrations (Fig. 2) were corrected for biovolume (pg chlorophyll per unit biovolume). In case of *Ochromonas* sp. no chlorophyll values for the LL‐treatment could be determined; the low material on the filter did not yield reliable values. For both species chlorophyll concentrations were inversely related with light (*O*. *perlata*: *F*_1,4_ = 15.190, *p* = 0.0176; *P*. *malhamensis*: *F*_1,8_ = 67.876, *p* < 0.001). In pigmented protists decreasing light leads to a higher investment in pigments, in order to efficiently use the available light (MacIntyre *et al*., 2002). In contrast, when *P*. *malhamensis* is rapidly growing on bacteria its growth rate may exceed the chlorophyll synthesis rate, resulting in reduced cellular chlorophyll content independent of light (Sanders *et al*., 1990; Holen, 2001). Although we did measure chlorophyll content only by the end of the experiment, when growth equilibrated, measurements of instantaneous chlorophyll fluorescence (*Ft*) throughout the experiment also indicate a reduction of chlorophyll content in the very early phase of the experiment (Fig. S1). That the chlorophyll concentrations of *P*. *malhamensis* by the end of the experiment were nonetheless inversely related with light hence may indicate that it was growing predominately photoautotrophically. Own preliminary experiments (data not shown) and previous studies showed that *P*. *malhamensis* is able to survive and grow in the dark, as long as prey is abundant (Caron *et al*., 1990; Sanders *et al*., 1990; Holen, 2001). Caron *et al*. (1990) and Sanders *et al*. (1990) suggested that *P*. *malhamensis* would ‘switch’ from phototrophic to heterotrophic growth when sufficient bacteria are available, and that phototrophy was of minor importance for its growth. Our study does not support this view, since the pronounced positive relationship between growth and light in the +DOC treatments indicate complimentary use of phagotrophy and photosynthesis. Within the here tested conditions this implies that light is a required source for *P*. *malhamensis* for optimal growth. In contrast to the aforementioned studies (Caron *et al*., 1990, Sanders *et al*. 1990, Holen, 2001) bacteria abundances in our study were much lower (see below), even when we consider prevalent differences in bacteria abundances between freshwater and marine environments. Though we were able to detect responses of the tested species to the glucose addition, particularly our results for *P*. *malhamensis* might be biased due to the overall low bacteria abundances.

In agreement with our third hypothesis, chlorophyll content was significantly higher (per light level) in *Ochromonas* sp., compared to *P*. *malhamensis* (*F*_1,15_ = 24.812, *p* = 0.0002). Chlorophyll content in *P*. *malhamensis* responded less to glucose addition, though overall glucose addition had no significant effect on cellular chlorophyll content in both species (*F*_1,15_ = 0.806, *p* = 0.3836). For *P*. *malhamensis* this may indicate that photosynthesis and energy acquisition through phagotrophy are uncoupled in this species and represent substitutable routes of resource acquisition. The fact that *Ochromonas* sp. was not able to grow under low light, irrespective of enhanced bacterial growth, suggests that photosynthesis and phagotrophy constitute complimentary routes of resource acquisition in this species. However, the lower, albeit not significantly different, cellular chlorophyll content of *Ochromonas* sp. in the treatments with glucose addition might point towards a low level of utilization of carbon acquired through bacterivory.

### Bacteria abundance

Abundances of bacteria (Fig. 2), measured at the end of the experiment, were independent of glucose addition in both species and hence represent the minimal prey requirements of each respective species under the applied light conditions. Thus, higher protist abundances in the +DOC treatments indicate higher bacterial production in these treatments. In the *P*. *malhamensis* treatments abundances of bacteria were significantly lower (*F*_1,19_ = 16.549, *p* = 0.0007), compared to the *Ochromonas* sp. treatments. This is in contrast to our first hypothesis that this presumably more heterotrophic mixotroph would require a higher minimal prey concentration, due to respirational losses. This could indicate that this species more efficiently uses bacterial prey, however, we cannot exclude that this is simply a result of different bacteria, potentially different in, e.g. food quality, present in the respective stock cultures, since no common prey was used. In the *Ochromonas* sp. treatments, bacteria abundances were inversely related to light (*F*_1,8_ = 13.146, *p* = 0.0067). This may indicate that this species is an obligate mixotroph, with increasing light availability grazing rates in this species increase. However, bacteria might also have been nutrient limited, with more severe limitation with increasing light, due to increasing uptake of nutrients by *Ochromonas* sp. with increasing light (see also section ‘Dissolved inorganic phosphorus’).

### Cellular C:P stoichiometry

Though the two tested species showed different responses to the applied environmental conditions, their cellular C:P stoichiometry remained, in agreement with our fourth hypothesis, rather stable across treatments and was similar to each other (Fig. 2). This validates that mixotrophy, irrespective of the prevailing physiological strategies used by mixotrophs in a system, indeed may enhance the transfer of energy and nutrients to higher trophic levels, resulting in more stable secondary production in plankton food webs (Katechakis *et al*., 2005; Ward and Follows, 2016; Moorthi *et al*., 2017).

## Conclusion

Only considering the results of this study, e.g. maximal growth rates (Fig. 2), *P*. *malhamensis* should be able to successfully compete with *Ochromonas* sp., at least under the tested conditions. However, assessing the gained specific competitive advantages of either mixotrophic strategy within complex communities based on single species experiments remains difficult. Generally, the plasticity in the nutrition of the facultative mixotroph *P*. *malhamensis*, demonstrated in this and previous studies, should enable growth under a wide range of environmental conditions. The nutrition of the possibly obligate mixotroph *Ochromonas* sp. appears to be much more constrained and should permit growth only in a narrow range of environmental conditions. This might seem disadvantageous at first; however, it was hypothesized that this strategy is likely efficient in stable environments with low resource availabilities, when the complimentary use of photosynthesis and phagotrophy presumably preserves resources and enables high resources use efficiency (Wilken *et al*., 2019). This is in line with the frequent observation of mixotrophic chrysophytes in oligotrophic systems (Ptacnik *et al*., 2008). The traits of the here tested species indicate differences in their niche differentiation. *Ochromonas* sp. may perform better in stable stratified surface water layers, where light is available, but prey abundances might be low. However, *P*. *malhamensis* should be able to also successfully compete in deeper water layers, where lower irradiance could limit growth of more light‐dependent mixotrophs, while facultative mixotrophs would benefit of higher bacterial production. This implies that the relative importance of mixotrophs with different physiological strategies in the same system is not only driven by prevailing environmental conditions but also by temporal changes of these conditions, e.g. seasonal changes in holomictic lakes. Facultative mixotrophy would be more advantageous under fluctuating conditions (mixing), while obligate mixotrophy could represent a specialization for stable stratified conditions, particularly during summer.

## Supporting information


**Appendix S1**. Supporting information.

## Data Availability

The data used for the statistics presented in the text and for the preparation of figures are available at https://datadryad.org (doi: 10.5061/dryad.ffbg79cx7).
