# Supplementary material for: Light‐dependent niche differentiation in two mixotrophic bacterivores
Source: Environ Microbiol Rep. 2022 May 4;14(4):530–7. doi: 10.1111/1758-2229.13071 (PMC9541266; doi:10.1111/1758-2229.13071)
Supplement: Supplementary file 1 — Appendix S1. Supporting information. [file EMI4-14-530-s001.docx]

**Supporting Information**

**Light dependent niche differentiation in two mixotrophic bacterivores**

**Robert Fischer^1*^, Julia Kitzwögerer^*^, Robert Ptacnik^1^**

^1^AquaScale, WasserCluster Lunz Biologische Station GmbH, Lunz am See, Austria

^2^ Austrian Biotech University of Applied Sciences, Biotech Campus Tulln, Tulln an der Donau, Austria

*** Correspondence:**

E-mail: [robert.fischer@wcl.ac.at](mailto:robert.fischer@wcl.ac.at)

**Keywords: mixotrophy, mixotrophic bacterivores, niche, traits, light, obligate mixotroph, facultative mixotroph, chrysophytes.**

**Species**

Two chrysophyte species, *Poterioochromonas malhamensis* (previously *Ochromonas* sp.; strain DS; e.g. Rothhaupt 1996) and *Ochromonas* sp*.*  (own isolate from Lake Lunz, available through the AquaScale lab), were used in this experiment. *P. malhamensis* is a well-studied chrysophyte species (e.g., Caron et al. 1990, Sanders et al. 1990, Rothhaupt 1996, Holen et al. 2001). It is described as primarily heterotrophic but capable of sustaining growth by photoautotrophy when prey concentrations limit heterotrophic growth (Caron et al. 1990). Species of the genus *Ochromonas* occur in marine and freshwater systems. Most studied *Ochromonas* species are considered to be primarily heterotrophic, however some species are unable to grow without light (e.g., Flöder et al. 2006, Fischer et al. 2017). Preliminary grazing experiments (data not shown) with the two species used here, indicated that both species reduced abundances of bacteria and cyanobacteria, i.e. both species grazed on bacteria sized prey. Stock cultures of both species were not axenic in terms of heterotrophic bacteria.

For the calculation of biovolume the dimensions of 30 or more cells of each species were measured at 400x magnification in samples fixed with a fixing agent consisting of glutaraldehyde and paraformaldehyde (final concentration 1%). Biovolume was then approximated using conversion factors for corresponding geometrical bodies of each respective species (spherical, for both species). The resulting biovolume of *Ochromonas* sp. was 117.07 µm³ and that of *P. malhamensis* was 158.55 µm³.

**Culture conditions**

Prior to the experiment, all organisms were pre-cultivated for several weeks. Algae were grown in a walk-in environmental chamber at a constant temperature of 18°C and a light:dark cycle of 12:12 hours. Light was supplied in a non-limiting irradiance of about 100 µmol photons m² s-1. Cultures were pre-grown on modified WEES medium (Kies 1967) based on sterile filtered lake water, without addition of soil extract. Nutrients were adjusted to 10% of the normal concentration, by diluting full WEES medium 1:10 with sterile filtered lake water (Lake Lunz; TP approx.5-8 µg L^-1^). Weekly dilution of the cultures ensured to keep cultures in exponential growth prior to the experiment. For the experiment the medium was further modified, no phosphorus source was added, hence only low amounts of inorganic phosphorous from the lake water (PO_4_: ~ 2 µg L^-1^) were available to the organisms.

**Experimental set up**

We used a continuous culture method, similar to a chemostat, the exponentially fed batch culture (for details see Fischer et al. 2014). A constant dilution rate of 0.1 d^-1^ was applied. The experiment was conducted in a walk-in environmental chamber at a constant temperature of 18°C. Light was supplied through a light panel consisting of a LED strip with three different LEDs (white, blue and red LEDs). The LED stripes were oriented horizontally in the back of three shelfs, at mid-height of the used culture flasks. Each LED stripe was individually controlled by a programmable LED time controller (Model TC420). By using LED as light source, we could manipulate light intensity independently from spectral composition.

In a two-factorial design, we manipulated the supply of light and glucose addition (+/-). Three different irradiance level were applied, 10 µmol photons m² s^-1^ (low light; LL), 60 µmol photons m² s^-1^ (intermediate light; IL) and 120 µmol photons m² s^-1^ (high light; HL), respectively. Each of the six resulting treatment combinations per species was replicated twice. The light:dark cycle was set to 12:12 hours, upon the onset and end of the light phase the light brightened to full irradiance and faded to darkness, respectively, in a period of one hour. Positions of each culture flask in the shelf were determined by irradiance measurements using a LI-COR LI 1400. The attenuation of about 6 % of the culture flask´s material was taken into account.

Tissue culture flasks (Cellstar, Greiner bio-one) with a volume of 650 mL were used for the experiment. Initial volume in each flask was 300 mL. Starting cell densities of *O. perlata* were ca. 4000 cells mL^-1^ and for *P. malhamensis* ca*.*1000 cells mL^-1^. To half of the flasks at each irradiance we added 10 µL of a glucose solution (stock solution: 10 mg L^-1^) at the start of the experiment and then every other day (after sampling), in order to enhance bacterial growth. We assumed both species were able to prey on the bacteria present in the respective non-axenic stock cultures.

**Sampling & Analyzes**

The duration of the experiment was 20 days. The experiment was terminated after instantaneous chlorophyll fluorescence values, measured via hand-held PAM fluorometer (AquaPen-C 100, Photon Systems Instruments, Czech) in most cultures did not chang for three consecutive samplings (data not shown). Instantaneous chlorophyll fluorescence (*Ft*) was measured upon every sampling, after keeping samples in the dark for at least 30 minutes. Cell densities were measured every other day using a flow cytometer (Cytoflex). Manual gating was used to detect populations of *O. perlata* and *P. malhamensis*, respectively (detection: 690 ± 50 nm versus 660 ± 20 nm). At the end of the experiment, samples were taken to measure dissolved and particulate nutrients as well as chlorophyll-a. For particulate fractions, samples were filtered on pre combusted and acid washed GF/F filters (Whatman). Separate filters were prepared for C vs. P. Prior to measurements, C and P filters were dried for at least 48 h in a drying chamber at 60°C and stored until analysis. Particulate C was measured with a CHN analyzer (Flash EA 1112; Thermo Fisher). Particulate P was measured as orthophosphate by a molybdate reaction after sulfuric acid digestion (Grasshoff et al., 1999, Solórzano and Sharp, 1980). Chlorophyll-a filters were stored in the freezer at -20 °C until measurement (Arar and Collins, 1997). Additionally, filtrates for measuring dissolved nutrients were collected and measured via continuous flow analysis (CFA, Alliance Instruments, Salzburg, Austria). For this experiment, only PO_4_ values were of interest, since all other nutrient were supplied in surplus. For the analysis of bacterial densities, 2 mL of sample was fixed with 20 µL fixing agent (1 % Paraformaldehyd and 10 % Glutaraldehyd) and stored at -20 °C until further analysis. Bacteria cell numbers were measured via flow cytometry. The samples were stained with SYTOX Green(Thermo Fisher Scientific), at least 10 minutes in the dark at room temperature. Bacteria were detected using manual gating after visual inspection of the dot plot of green fluorescence (detection: 525 ± 40) versus right angle side scatter (SSC).

**Statistics**

Protist and bacteria abundances, dissolved P concentrations, chlorophyll content (corrected for biovolume) and stoichiometric rates at steady state were tested for differences between species as well as effects of light and glucose addition by ANCOVA. Effects of light and glucose addition were also analyzed separately for each species by ANCOVA to assess their impact and interaction in each species individually. Where necessary, response variables were log-transformed or square root-transformed, respectively, to improve homoscedasticity. By fitting the time trends to daily intervals using generalized additive models, we could calculate daily net growth rates for the protists. As the protists did not experience grazing, we estimated their respective gross growth rates by adding the dilution rate to the net growth rates. All statistical analyses were done using R ver. 3.5.2 (www.r-project.org).

**References**

Arar, E. J., & Collins, G. B. (1997). Method 445.0: In vitro determination of chlorophyll a and pheophytin a in marine and freshwater algae by fluorescence. Washington, DC, USA: United States Environmental Protection Agency, Office of Research and Development, National Exposure Research Laboratory.

Rothhaupt, K. O. (1996). Laboratorary experiments with a mixotrophic chrysophyte and obligately phagotrophic and photographic competitors. Ecology, 77(3), 716-724. doi.org/10.2307/2265496

Caron, D. A., Porter, K. G., & Sanders, R. W. (1990). Carbon, nitrogen, and phosphorus budgets for the mixotrophic phytoflagellate Poterioochromonas malhamensis (Chrysophyceae) during bacterial ingestion. Limnology and Oceanography, 35(2), 433-443. doi.org/10.4319/lo.1990.35.2.0433

Sanders, R. W., Porter, K. G., & Caron, D. A. (1990). Relationship between phototrophy and phagotrophy in the mixotrophic chrysophyte Poterioochromonas malhamensis. Microbial Ecology, 19(1), 97-109. doi.org/10.1007/BF02015056

Flöder, S., Hansen, T., & Ptacnik, R. (2006). Energy–dependent bacterivory in Ochromonas minima–A strategy promoting the use of substitutable resources and survival at insufficient light supply. Protist, 157(3), 291-302. doi.org/10.1016/j.protis.2006.05.002

Fischer, R., Giebel, H. A., Hillebrand, H., & Ptacnik, R. (2017). Importance of mixotrophic bacterivory can be predicted by light and loss rates. Oikos, 126(5), 713-722. doi.org/10.1111/oik.03539

Holen, D. A. (2001). The effects of heterotrophy on chlorophyll a and photosynthesis in a mixotrophic chrysophyte. NOVA HEDWIGIA BEIHEFT, 122, 107-118.

Kies, L. (1967). Über Zellteilung und Zygotenbildung bei Roya obtusa (BREB.) WEST et WEST. Mitt. Staatsinst. Allg. Bot. Hamburg, 12, 35-42.

Fischer, R., Andersen, T., Hillebrand, H., & Ptacnik, R. (2014). The exponentially fed batch culture as a reliable alternative to conventional chemostats. Limnology and Oceanography: Methods, 12(7), 432-440. doi.org/10.4319/lom.2014.12.432

Grasshoff K., Kremling K. & Ehrhardt M. (Eds.), 1999. Methods of seawater analysis, 600 p. New York: Wiley.

Solórzano, L., & Sharp, J. H. (1980). Determination of total dissolved phosphorus and particulate phosphorus in natural waters 1. Limnology and Oceanography, 25(4), 754-758. doi.org/10.4319/lo.1980.25.4.0754


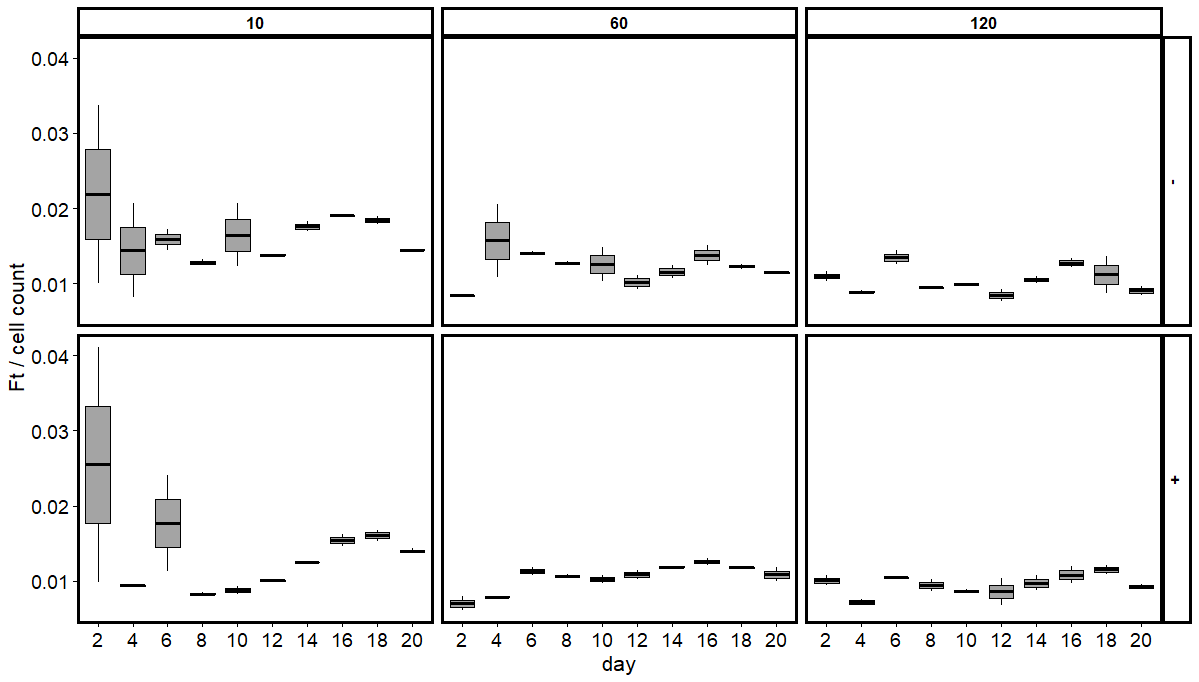


Figure S1: Instantaneous chlorophyll fluorescence of *Poterioochromonas malhamensis* , corrected for abundance (cells mL^-1^), over time, in dependence of light and glucose addition (+DOC).


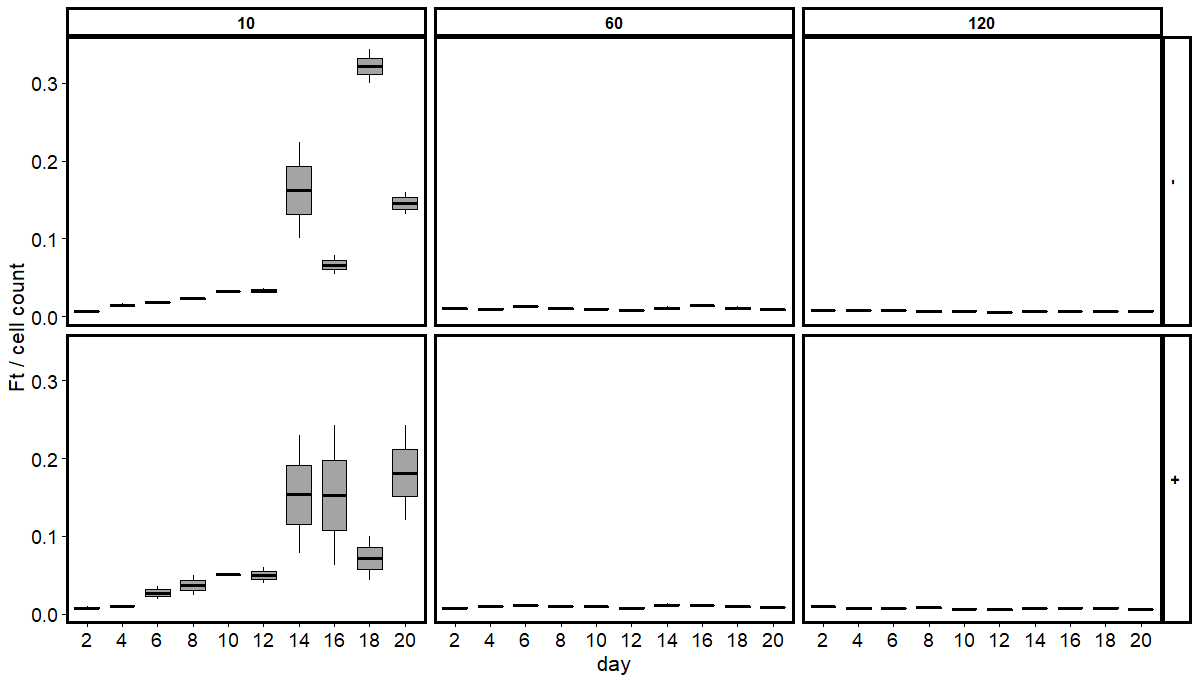


Figure S1: Instantaneous chlorophyll fluorescence of *Ochromonas* sp. , corrected for abundance (cells mL^-1^), over time, in dependence of light and glucose addition (+DOC).
